# Diseases of the Jaw

**Published:** 1869-06

**Authors:** Thomas Waterman

**Affiliations:** Boston.


### ARTICLE. X.

### *Diseases of the Jaw.*

By Thomas Waterman, M.D., Boston.

1.—*Naso-pharyngeal Polypus. Extirpation preceded by Temporary Displacement of the Superior Maxilla.*—B.F.F., æt. 39. A polypus of the left nasal fossa has been steadily growing for four years. It is visible just within the anterior nares, can be felt behind the soft palate, and can be seen by raising the palate with a spatula. It is hard and firm to the touch, does not readily bleed, and is not accompanied by deafness. Its point of origin is plainly from the posterior part of the nasal fossa. The left side of the nose is distended by the polypus, giving to the face that characteristic expression accompanying similar growths.

In view of the size and obviously fibrous character of the growth, as well as its inevitable tendency, no other mode of removal than its direct excision at its point of origin seemed admissible, and this could be effected only by removing the upper jaw in a way and to an extent sufficient to expose the whole nasal fossa.

*Operation.*—A vertical incision was made from the nostril through the upper lip, and the cheek dissected up freely from the bone. The maxillary bone was then sawed horizontally across just below the floor of the orbit, from its outer border to the nasal fossa; the intermaxillary suture was divided by bone forceps, the mucous membrane of the hard palate having been previously incised along the median line. A broad chisel inserted into the cut made by the saw depressed the bone, fracturing it posteriorly at its connection with the palate bones. By this deplacement and without any further detachment, the origin of the polypus could be easily reached; the growth, which consisted of many firm lobules, was cut and torn away from the sphenoidal bone into the cells of which it had penetrated. The point from which it grew was then thoroughly swabbed with Squibb's liquor ferri sulphatis, care being taken not to bring it in contact with the cut surfaces of the displaced bone. No ligatures were required. The polypus being removed, the bone was replaced and held in position by a silver wire twisted around the incisors on either side of the median section, a cork wedge was placed between the posterior molars, and the lower jaw bandaged firmly against the upper.

On the ninth day after the operation the patient was out of doors, on the eleventh an attack of erysipelas confined him to his bed again for a fortnight, but with no detriment to the progressing union of the jaw, which was perfected sufficiently to permit the removal of the wire on Oct. 18th, five weeks from the date of operation (Sept. 14th). On Oct. 28th, he was discharged from the Hospital by his own request. He had been able for ten days or a fortnight to chew meat with the teeth of the affected side, so firm was the union, and there was no deformity of the face, the trifling scar of the lip being invisible under his moustache. Two or three days before he left, a triangular piece of dead bone, about one inch long and one-third of an inch broad, came out through his nose. It appeared to be a portion of the palatal process of the superior maxilla.

Temporary resections, or osteoplastic resections, as they are termed in Europe, are characterized by the displacement of a bone still partially held in place by the soft parts; and by replacement of the bone, which has been thus rendered movable, as soon as the extirpation of the tumor is complete. The traces of the methods by which the surgeon obtained access to the tumor are thus effaced.*

* Rapport sur les Progres de la chirurgie, Paris, 1867; in this work a history of the operation of temporary displacement of the upper jaw may be found, also in the Sydenham Year-Book of Medicine and Surgery, 1865, pp. 271 and 295.

The result of these procedures, as well as that of complete excision of the upper jaw, illustrates the extent to which operations may be successfully practiced upon the bones of the face which protect and enclose important parts, but are independent of vital organs. The particular operation under consideration is undoubtedly a valuable resource in many cases hitherto requiring a still severer mutilation, but as shown in the case next reported it does not admit of universal application. The improvements of modern dentistry are available for the diminution of much of the deformity entailed by the entire removal of the superior maxilla ; an artificial jaw of vulcanite not only restores the dental arch, but obviates the unsightly falling in of the cheek usually consequent upon this operation.

II.—*Pharyngeal Tumor. Extirpation preceded by Resection of Superior Maxilla.*—J. S. I., æt. 33. Fourteen months since a tumor of the size of a hen's egg, springing from the vicinity of the left tonsil, was removed by the ecraseur. It was thought at the time to be probably malignant, but his recovery from the operation was rapid, and on examining his throat no trace of its existence or point of implantation can now be seen. Within two months his ability to blow air through the left nostril has gradually ceased, at present it is entirely obstructed. The right nostril is also partly obstructed, and to an increasing extent. His deglutition as well as respiration is difficult. On introducing the finger behind the soft palate a growth having a broad surface of origin from the basilar process of the spheno-occipital bone fills the left half of the space between the base of the skull and the posterior nares. The finger can with difficulty be swept around the tumor on account of the small space unoccupied by it, but its attachment and the constriction of its base can readily be felt. No part of the tumor enters the nasal cavities, it cannot be seen from the anterior nares, nor is there any external or visible deformity. The tumor is symmetrical in shape, bleeds on touch as it also does spontaneously or from sneezing, is firm and hard, though friable, and is not painful or sensitive. There is no enlargement of the lymphatic glands. It was not inspected with the aid of the rhinoscope. As the disease was inaccessible for thorough and complete removal, without the excision of the left superior maxillary bone, neither the division of the soft palate (*Manne*) nor the partial removal of the hard palate (*Nelaton*) offering any chance of getting at the tumor, that operation was performed Oct. 12th, by the method usually described as of Velpeau. Through the aperture thus afforded the tumor was rendered visible as well as accessible, presenting a round convex mass an inch and a half in diameter furrowed by the septum of the nostrils. It was removed with the aid of curved scissors, the bone from which it grew was cut away with the gouge, although not apparently diseased, and the surface thus denuded, as well as the soft parts adjoining, were swabbed with Sqibb's liquor ferri subsulphatis. Two or three ligatures only were requirad.

The tumor under the microscope proved to be glandular rather than malignant. According to Dr. C. Ellis, " it was composed of rather small nuclei, with pale nucleoli somewhat larger than those usually found in glandular growths, but resembling them in other respects. A few doubtful lobules and some fragments of lobules were also seen. There were also found some fibrous tissue and a few minute blood-vessels. Very few, if any cells, and those of small size."

On the third day from the operation the stitches were removed from the incision in the cheek; on the ninth the patient sat up, and on the fourteenth he was discharged.

In February last he visited the Hospital wearing an artificial jaw which, exclusive of the palatine arch, was not more than one inch in diameter, so completely had the cavity left by the operation filled up. The scar on the cheek was invisible beneath his whiskers, there was no falling in of the cheek, dropping of the lower eye-lid, nor paralysis of the face. The tone of his voice was not noticeably nasal, and there had been no recurrence of the tumor.

III.—*Hypertrophy of Gums. Partial Resection of Superior Maxilla.*—M. A. S, a young woman of average mental capacity, æt. 27. She has never been in good health. Her mother and her nurse say that the disease of which she is the subject is not congenital, but ever since the patient herself can remember she has been asked "what is the matter with your gums?" She has repeatedly had abscesses about the mouth, gum-boils, catarrh, and suffered most of her life from thick speech, deafness, difficult deglutition and dull pain in the jaws.

On examination the gums are seen to be hypertrophied along each side of the dental arches, not uniformly, but more prominently at some points than at others. The principal outgrowths are in front of the canine aud incisor teeth in the upper jaw; in the lower jaw they occupy the place of the molar teeth on both sides. Iu the palatine arch of the superior maxillary bones two projecting excrescences, having their attachment anteriorly, pass backward, concealing the soft palate; in the cleft between them the uvula can be seen. Ou passing the finger into this cleft it can be swept around slightly, the soft palate and a small part of the hard palate not being connected with the growth. These excrescences feel quite hard and non-elastic. The portions which project backward are somewhat movable, and can be pressed up so as to touch the palate.

At various times several teeth have been extracted, and the patient thinks that this has caused the growth to shrink somewhat, but the changes have been slight during the last eight years.

On the 26th of June all the teeth of the upper jaw were extracted, and and at the same time those portions of the excrescences of the upper jaw which concealed the soft palate were sliced off. The patient was discharged on the 3d. of July, and re-entered the Hospital Oct. 8th. The disease in the meantime had remained quiescent.

Oct. 9th, the whole of the outgrowths were removed with the gouge, and the dental border of the superior maxilla sawed off. The wounds healed rapidly, and on the 21st of Oct. the patient was discharged, with the cut surfaces granulating in a healthy manner.

The rarity of the disease has led me to report this case, the interest of which centres in the peculiarity and infrequency of such an hypertrophy, rather than in the result of the operation.

I find but three recorded cases of this disease, one by Prof. Gross,* one by Mr. Pollock,† and a third by Mr. Heath,‡ occurring under the care of Mr. Erichsen, in Univ. Coll. Hosp. In the first two cases the disease was congenital, and returned to some extent after removal. A very remarkable specimen of this disease presented itself in the person of a female of feeble intellect, covered with a remarkable hairy growth, who was exhibited by a showman in this city some ten years ago under the name of "Bear Woman." The hypertrophy of the gums was even more conspicuous than in the recorded cases. It is a little singular that Mr. Pollock's case was characterized by an extraordinary pilous development, and the patient a subject of epilepsy. Dr. Gross's patient was a stunted and feeble-minded boy.

Under the microscope the disease presented a purely fibrous growth, without myeloid cells, distinguishing it from epulis, with which, however, it was little likely to be confounded, neither the general aspect nor the mode of its growth bearing resemblance to the distinct masses and interdental origin of that affection.

The gross appearances of hypertrophied gums resemble the disease called lampas, occurring in the horse. The latter however, is an *inflammation* of the gums, propagated to the bars of the roof of the mouth, and rising to a level with and even beyond the teeth. It usually subsides without treatment, or only requires slight scarification.

IV.—*Tumor of the Lower Jaw from a misplaced Wisdom Tooth. Operation for its removal.*—A colored woman,

---

* Gross's System of Surgery, 2d edition, Vol. II., p. 534, fig, 330.
† Holmes's System of Surgery, Vol. IV., p. 18.
§ Injuries and Diseases of the Jaws, London, 1868, p. 189.

æt. 41, ten years ago noticed an enlargement of the lower jaw on the left side, near the angle in the region usually occupied by the molar teeth. No permanent molars had ever appeared on that side, and it was the patient's conviction that there never had been any deciduous molars. The enlargement of the jaw was principally of the alveolar border, and this finally grew to such a degree as to prevent bringing the teeth together. Under these circumstances, five years ago a portion of the tumor cartilaginous in density was shaved off. A new growth gradually replaced what was removed, and there is now an enlargement of the entire bone, firm, inelastic, slightly irregular in outline, sensitive on the inside to touch, and whenever hard morsels are bitten upon. It is hardly of sufficient size to be visible from the outside, but can readily be felt, and it projects inwards about to the same extent. The jaw is perhaps double its natural thickness. For the last six months the tumor has been the centre of a radiating neuralgic pain constantly present, and so severe as to make the patient willing to undergo any operation likely to give her relief.

Removal of a portion of the continuity of the jaw being attended by disability and disfigurement, it was thought best to perform a temporizing operation, and excise so much of the tumor as could be from the inside of the mouth. In chiselling away the bone, which was dense and vascular, a well-formed wisdom tooth was found impacted in the jaw bone in a horizontal position. As this was deemed to have been the source of all the suffering as well as to constitute the tumor, no further steps were taken toward its more thorough extirpation. The operation was followed by complete disappearance of the pain. The wound rapidly granulated, and at the end of three weeks the patient was discharged at her own request.

The crown of the tooth removed was found to be enveloped by the membranous sac originally lined with enamel pulp, which having fulfiled its function had become detached from the surface of the enamel, and now remained as a capsular investment of that portion of the tooth. The sac thus formed was not distended with serous fluid into a "dentigerous cyst," as occasionally occurs, and an instance of which was reported in 1863,* but retained its original proportions. The case must therefore be looked upon merely as one of impacted misplaced tooth, and the specimen is interesting from its deep-seated position, and as exhibiting the *pathogenesis* rather than the *pathology* of dentigerous cysts, in a manner all the more satisfactory from the rarity with which an opportunity is afforded for their study.

The subject of dentigerous cysts has been treated of at length by Mr. Salter.†

(The preceding cases of more than usual interest occurred in 1867, at the Massachusetts General Hospital.)—*Boston Med. & Surg. Journal.*

* Trans. Boston Soc. for Med. Improvement. Vol. V. ,p 100.
† Guy's Hosp. Reports, Vol. V., 3d Series, p. 319 and Holmes's Surgery, Vol. IV., p. 32.

